# Maturation of the Mitochondrial Redox Response to Profound Asphyxia in Fetal Sheep

**DOI:** 10.1371/journal.pone.0039273

**Published:** 2012-06-15

**Authors:** Paul P. Drury, Laura Bennet, Lindsea C. Booth, Joanne O. Davidson, Guido Wassink, Alistair J. Gunn

**Affiliations:** 1 Fetal Physiology and Neuroscience Group, Department of Physiology, The University of Auckland, Auckland, New Zealand; 2 Howard Florey Institute, University of Melbourne, Melbourne, Victoria, Australia; 3 Starship Children's Hospital, Auckland, New Zealand; Université de Montréal, Canada

## Abstract

Fetal susceptibility to hypoxic brain injury increases over the last third of gestation. This study examined the hypothesis that this is associated with impaired mitochondrial adaptation, as measured by more rapid oxidation of cytochrome oxidase (CytOx) during profound asphyxia. Methods: Chronically instrumented fetal sheep at 0.6, 0.7, and 0.85 gestation were subjected to either 30 min (0.6 gestational age (ga), n = 6), 25 min (0.7 ga, n = 27) or 15 min (0.85 ga, n = 17) of complete umbilical cord occlusion. Fetal EEG, cerebral impedance (to measure brain swelling) and near-infrared spectroscopy-derived intra-cerebral oxygenation (ΔHb = HbO_2_ – Hb), total hemoglobin (THb) and CytOx redox state were monitored continuously. Occlusion was associated with profound, rapid fall in ΔHb in all groups to a plateau from 6 min, greatest at 0.85 ga compared to 0.6 and 0.7 ga (p<0.05). THb initially increased at all ages, with the greatest rise at 0.85 ga (p<0.05), followed by a progressive fall from 7 min in all groups. CytOx initially increased in all groups with the greatest rise at 0.85 ga (p<0.05), followed by a further, delayed increase in preterm fetuses, but a striking fall in the 0.85 group after 6 min of occlusion. Cerebral impedance (a measure of cytotoxic edema) increased earlier and more rapidly with greater gestation. In conclusion, the more rapid rise in CytOx and cortical impedance during profound asphyxia with greater maturation is consistent with increasing dependence on oxidative metabolism leading to earlier onset of neural energy failure before the onset of systemic hypotension.

## Introduction

The mammalian fetus has a remarkable ability to adapt to and survive far more prolonged periods of asphyxia than adults. During fetal life, cardiovascular tolerance to severe asphyxia is typically maximal near-midgestation, corresponding with the time of maximal levels of cardiac glycogen [1], and falls progressively towards term [2]. Neurological tolerance appears to broadly parallel cardiac survival. For example, near-term fetal sheep develop selective neural injury after 10 min of complete umbilical cord occlusion [3], [4], with much greater injury and reduced survival with longer insults of up to 15 min [5], [6]. In contrast, 0.6 gestation fetal sheep develop little or no injury even after 20 min of occlusion [3], [7] and severe, subcortical neural injury requires 30 min of complete occlusion [8].

Potentially then, greater susceptibility to asphyxial neural injury with increasing gestation could be related to either a change in the intrinsic tolerance of the developing brain or to the more rapid onset of profound hypotension and hypoperfusion near-term [2]. In the fetal sheep basal cerebral blood flow and oxygen consumption per 100 g weight increase towards term [9], [10], consistent with greater basal neural aerobic dependence that would increase the vulnerability of the brain to asphyxia in late gestation. A similar increase in oxygen consumption and oxygen delivery with increasing gestation is seen in the guinea pig [11], and there is evidence that the preterm brain generates a greater proportion of ATP through non-oxidative metabolism compared to at term [12], [13]. Further, in human infants the ratio of phosphocreatine (PCr) to inorganic orthophosphate increases between 28 and 42 weeks gestation, suggesting increasing basal metabolism [14].

More important, effective adaptation to asphyxia must involve the maximum possible reduction in non-essential energy-utilizing processes, particularly synaptic transmission [15]–[17]. This initial suppression in brain activity is actively mediated by increased levels of inhibitory neuromodulators including adenosine [17], [18], which has been termed ‘adaptive hypometabolism’ [19]. There is limited information on how the ability to suppress brain metabolism changes with advancing gestation, however, the minimum energy requirements for essential cell survival seem to be lower earlier in gestation [13], [20], likely in part related to reduced dendritic complexity [21]. If this hypothesis is correct, then we may predict that high energy metabolites would be depleted more rapidly during asphyxia closer to term.

These changes can be indirectly monitored by using near-infrared spectroscopy (NIRS) to continuously measure changes in the oxidized state of the CuA moiety of cytochrome oxidase (CytOx) [22]. CytOx is linked to complex IV as the terminal electron acceptor in the electron transport chain. When CytOx has electrons available to be donated it is reduced. Conversely, when all the electrons have been donated to oxygen to form water with H^+^, creating the transmembrane gradient that drives ATP production, CytOx is oxidized [23]. What determines its redox state then is the balance between electrons being passed down the electron chain and the rate at which they are donated to oxygen. Intracellular ATP levels are buffered by PCr [23], but this pool is very rapidly depleted. Thus, a relative increase in oxidized CytOx suggests either more rapid consumption of electrons to produce ATP (as seen in skeletal muscle during intense exercise [24]), or reduced availability of reducing equivalents from the electron transport chain [25], [26].

In the context of the very rapid, profound de-oxygenation during complete umbilical cord occlusion, an increase in oxidized CytOx must reflect a marked depletion of reducing equivalents transferred from the tricarboxylic acid cycle, rather than increased donation of electrons to oxygen. Consistent with this, we have previously shown that during asphyxia moderately preterm (0.7 gestation) fetal sheep show a rapid initial increase in oxidized CytOx measured using NIRS [27]. Strikingly, there was a substantial delay before CytOx reached peak values, raising the possibility that adaptive hypometabolism may be more effective in preventing injury in the preterm brain than at term, however, this has not been directly assessed.

In the present study we tested the hypothesis that profound asphyxia would be associated with a more rapid initial increase in oxidation of CytOx with greater maturation, before the onset of systemic hypotension. Changes in mitochondrial redox state, cerebral oxygenation, EEG power, cortical impedance (a measure of cell swelling [28]) and carotid blood flow (as an index of global cephalic perfusion [17], [29]–[32]) were measured during complete umbilical cord occlusion at 0.6, 0.7, and 0.85 gestation. These ages are broadly equivalent to the neural maturation of the human fetus at 26–28-wk, 28–32-wk, and 40-wk gestation, respectively [33], [34]. In separate groups we also assessed changes in local cortical blood flow at 0.7, and 0.85 gestation using laser Doppler [17].

## Methods

### Surgical procedures

All procedures were approved by the Animal Ethics Committee of the University of Auckland. 38 singleton Romney/Suffolk fetal sheep were operated on at 84–86 d (0.6 of gestation, n = 6), 96–99 d (0.7 of gestation, n = 22 with NIRS optodes *plus* n = 5 with laser Doppler probes, as below) and 118–125 d (0.85 of gestation, n = 9 with NIRS optodes *plus* n = 8 with laser Doppler probes) gestational age (term = 147 days). Food, but not water was withdrawn 18 h before surgery. Ewes were given 5 ml of procaine penicillin (250 000 IU) with dihydrostreptomycin (250 mg ml−1, Stockguard Laboratories Ltd, Hamilton, New Zealand) intramuscularly for prophylaxis 30 min prior to the start of surgery. Anesthesia was induced by i.v. injection of Aflaxan (3 mg kg−1; Alphaxalone, Jurox, Rutherford, NSW, Australia), and general anesthesia maintained using 2–3% isoflurane in O_2_. Under anesthesia a 20-g i.v. catheter was placed in a maternal front leg vein and the ewes were placed on a constant infusion saline drip to maintain maternal fluid balance. Ewes were ventilated if necessary and the depth of anesthesia, maternal heart rate and respiration were constantly monitored by trained anesthetic staff.

All surgical procedures were performed using sterile techniques [30]. Following a maternal midline abdominal incision and exteriorization of the uterus and either the top or bottom half of the fetus, catheters were placed in the left fetal femoral artery and vein, right brachial artery and vein, and the amniotic sac. An ultrasonic blood flow probe (size 3S; Transonic Systems Inc., Ithaca, NY, USA) was placed around the left carotid artery to measure carotid artery blood flow (CaBF) as an index of global cephalic blood flow. Two pairs of EEG electrodes (AS633-5SSF, Cooner Wire Co., Chatsworth, CA, USA) were placed through burr holes on the dura over the parasagittal parietal cortex (5 mm and 10 mm anterior to bregma and 5 mm lateral) and secured with cyanoacrylate glue. To measure cortical impedance a third pair of electrodes was placed over the dura 5 mm lateral to the EEG electrodes. A reference electrode was sewn over the occiput. A pair of electrodes were sewn over the fetal chest to measure the fetal ECG. An inflatable silicone occluder was placed around the umbilical cord of all fetuses (In Vivo Metric, Healdsburg, CA, USA). A flexible fiber optic probe (diameter ∼400 µm) containing emitting and receiving laser Doppler channels was placed in the right parietal cortex approximately 5 mm lateral to the midline and 15 mm anterior to bregma, to a depth of 5 mm below the dura, in the grey matter of the cortex of 0.7 and 0.85 gestation fetuses only (Oxford Optronix Inc., Oxford, UK) [17]. Two small flexible fiber optic probes, used for the near infrared spectroscopy recordings, were placed biparietally on the skull 3.0 to 3.5 cm apart, 1.5 cm anterior to bregma, and secured using rapid setting dental cement (Rocket Red, Dental Adventures of America, Inc., Anaheim, CA, USA) [27], [35]. NIRS and laser Doppler were recorded in separate groups of animals as light from the laser Doppler interferes with NIRS measurements. All fetal leads were exteriorized through the maternal flank and a maternal long saphenous vein was catheterized to provide access for post-operative care and euthanasia. Antibiotics (80 mg Gentamicin, Rousell, Auckland, New Zealand) were administered into the amniotic sac prior to closure of the uterus.

Post-operatively all sheep were housed in separate metabolic cages with access to water and food *ad libitum*, together in a temperature-controlled room (16±1°C, humidity 50±10%) with a 12 h light/dark cycle. A period of 5 days post-operative recovery was allowed before experiments commenced, during which time antibiotics were administered to the ewe i.v. (4 days 600 mg Benzylpenicillin Sodium; Novartis Ltd, Auckland, New Zealand, and 2 days 80 mg Gentamicin). Fetal catheters were maintained patent by continuous infusion of heparinized saline (20 U/ml at 0.2 ml/h) and the maternal catheter maintained by daily flushing.

### Experimental Recordings

Fetal mean arterial blood pressure (MAP), corrected for maternal movement by subtraction of amniotic fluid pressure (Novatrans II, MX860; Medex Inc., Hilliard, OH, USA) [36], ECG, EEG, and impedance were recorded continuously. The blood pressure signal was collected at 64 Hz and low pass filtered at 30 Hz. The EEG signal was high-pass filtered at 1.6 Hz and low-pass filtered at 50 Hz, then stored for offline analysis at a sampling rate of 256 Hz. The high-pass filter had a first-order roll-off of 6 dB per octave, thus attenuating but not removing frequencies below this. Total EEG power (µV^2^) was calculated from the intensity spectra by fast Fourier transform of the EEG on sequential epochs, using a 10 second Hanning-window to minimize spectral leakage [37]. Cerebral impedance was calculated as previously described [28]. The impedance of a tissue rises concomitantly as cells depolarize and fluid shifts from the extracellular to intracellular space, and thus impedance is a measure of cytotoxic edema. Data were collected by computer and stored to disk for off-line analysis.

### Experimental protocol

Experiments were conducted at 88–90 d (0.6), 101–104 d (0.7), and 121–128 d (0.85) gestation. Fetal asphyxia was induced by rapid inflation of the umbilical cord occluder for 30 min in the 0.6 group, 25 min in the 0.7 group and 12–15 min in the 0.85 group with sterile saline of a defined volume known to completely inflate the occluder and totally compress the umbilical cord, as determined in pilot experiments with a Transonic flow probe placed around an umbilical vein [30]. Successful occlusion was confirmed by observation of a rapid onset of bradycardia with a rise in MAP, and by pH and blood gas measurements. If fetal blood pressure fell below 8 mmHg in the 0.6 and 0.7 groups or below 12 mmHg in the 0.85 group then the occlusion was ended immediately. The duration of occlusions were chosen to represent acute, severe, near-terminal insults, associated with severe neuronal loss. All occlusions were undertaken between 0900 and 1000 h. After release of the umbilical cord occluder fetuses were allowed to auto-resuscitate. If fetal heart rate (FHR) was not above 100 bpm within 1 min of occlusion release then 0.1–0.3 ml/kg of 1/10000 adrenaline (DBL, Hospira, Auckland, New Zealand) was administered via the brachial vein. If no response was observed then the ewe was euthanized following fetal death.

Fetal arterial blood was taken at 15 min prior to asphyxia (baseline) and at appropriate early and late time points during asphyxia: 5 and 25 min in the 0.6 group, 5 and 17 min in the 0.7 group, and 2 and 12 min in the 0.85 group during asphyxia for pH and blood gas determination (Ciba-Corning Diagnostics 845 blood gas analyzer and co-oximeter, MA., USA) and for glucose and lactate measurements (YSI model 2300, Yellow Springs, Ohio, USA). At the end of the experiment ewes and fetuses were killed by an intravenous overdose of pentobarbitone sodium (9 g) to the ewe (Pentobarb 300; Chemstock International, Christchurch, New Zealand).

### Near-infrared spectroscopy (NIRS) measurements

Concentration changes in fetal cerebral deoxyhemoglobin ([Hb]), oxyhemoglobin ([HbO_2_]) and oxidised cytochrome oxidase [CytOx] were measured using a NIRO-500 spectrophotometer (Hamamatsu Photonics KK, Hamamatsu City, Japan) and data recorded by computer for off-line analysis. As described previously [38], near-infrared light, at four different wavelengths between 775 and 908 nm, was carried to the fetal head through a fiber optic bundle. Emerging light was collected by the second optode and transmitted to the spectrophotometer. Changes in the cerebral [HbO_2_], [Hb] and [CytOx] were calculated from the modified Lambert-Beer law using a previously established algorithm which describes optical absorption in a highly scattering medium [39]. These NIRS measures are expressed as relative change from zero. Standardization of the distance between the optodes and fixation of the optodes to the surface of the skull by dental cement were used to reduce signal variability within and between subjects in this study.

Two key parameters were calculated: total hemoglobin ([THb]): the sum of [HbO_2_] and [Hb], and [ΔHb]: the difference between [HbO_2_] and [Hb]. THb is related to cerebral blood volume (CBV) by the cerebral hematocrit: CBV = [THb]/(HR) where H is the arterial hematocrit and R is the cerebral-to-large vessel hematocrit ratio, assumed to be 0.69 [39]. THb is a reliable index of the hemoglobin content of the brain, and thus of CBV, given stable blood hemoglobin and hematocrit [40]. ΔHb is a measure of total intravascular oxygenation in the brain [41].

### Data analysis

Off-line analysis of the physiological data was performed using customized Labview programs. Data were analyzed using JMP 8.0 (SAS Institute, Cary, North Carolina, USA) and SPSS for windows (SPSS, Chicago, Il, USA). For between group comparisons analysis of variance for repeated measures was performed. When statistical significance was found one-way analysis of variance with post-hoc LSD tests was used to compare selected time points. Within subjects regression was performed to compare laser Doppler cortical blood flow and carotid artery blood flow using the Bland-Altman method [42]. Statistical significance was accepted when p<0.05. Data are mean±SEM.

## Results

Umbilical cord occlusion was associated with a progressive, profound hypoxemia, hypercapnia and acidosis; hemoconcentration developed at all ages (Table 1).

**Table 1:** Blood gases, acid-base status, glucose and lactate were measured on fetal arterial blood taken at 15 min prior to asphyxia (baseline) and at an early and late time points during asphyxia: 5 and 25 min at 0.6 gestation, 5 and 17 min at 0.7 gestation, and 2 and 12 min at 0.85 gestation during asphyxia, Hb: hemoglobin concentration; Hct: hematocrit; O2ct: oxygen concentration; BE: base excess.

		Baseline	2/5 min	12/17/25 min
**pH**	0.6	7.38±0.01†	7.06±0.01^#^ ‡	6.77±0.02^#^ ‡
	0.7	7.36±0.00†	7.05±0.01^#^ ‡	6.83±0.01^#^ ‡ §
	0.85	7.39±0.01	7.24±0.02#	6.91±0.02#
**paCO_2_**	0.6	45.0±0.7	89.5±2.8^#^ ‡	152.4±5.2^#^ †
(mmHg)	0.7	49.1±0.9§	99.7±2.8^#^ ‡ §	146.6±3.3^#^ †
	0.85	51.7±1.0§	68.1±2.8#	132.5±3.0#
**paO_2_**	0.6	24.2±0.6†	6.6±0.9#	8.1±0.9^#^ †
(mmHg)	0.7	22.7±0.8	5.9±0.5#	8.7±0.6^#^ ‡
	0.85	20.3±0.6	6.6±0.6#	5.4±0.7#
**Hb**	0.6	8.2±0.2‡	8.9±0.3^#^ †	8.4±0.3‡
(g.dL^−1^)	0.7	8.6±0.2†	9.5±0.3#	9.2±0.2^#^ ‡
	0.85	9.9±0.4	10.5±0.6*	10.9±0.5#
**Hct**	0.6	23.9±0.6‡	26.0±0.8^#^ †	24.7±0.9‡
	0.7	25.5±0.6†	28.2±0.8#	27.0±0.7^#^ ‡
	0.85	29.2±1.3	30.8±1.7*	32.1±1.4#
**O_2_ct**	0.6	3.5±0.1	0.4±0.1^#^ †	0.4±0.0#
(mmol.L^−1^)	0.7	3.4±0.1	0.4±0.0#	0.5±0.0#
	0.85	3.5±0.2	0.5±0.0#	0.4±0.0#
**HCO_3_^−^**	0.6	25.2±0.5‡	18.1±0.4^#^ ‡	12.2±0.4^#^ ‡
(mmol.L^−1^)	0.7	25.7±0.4‡	18.6±0.5^#^ ‡	15.9±0.9#
	0.85	28.6±0.4	25.0±0.6#	19.4±1.7#
**BE**	0.6	1.2±0.5‡	−6.2±0.6^#^ ‡	−14.1±0.5^#^ ‡
(mmol.L^−1^)	0.7	2.6±0.4‡	−5.5±0.7^#^ ‡	−11.4±0.5^#^ ‡ §
	0.85	4.5±0.5	0.3±0.6#	−8.9±0.5#
**Lactate**	0.6	0.74±0.07	3.93±0.21^#^ ‡	7.46±0.30^#^ ‡
(mmol.L^−1^)	0.7	0.99±0.21	3.98±0.15^#^ ‡	6.97±0.20^#^ †
	0.85	1.16±0.11	2.09±0.17#	5.92±0.29#
**Glucose**	0.6	1.11±0.09†	0.46±0.11#	0.49±0.13^#^ †
(mmol.L^−1^)	0.7	1.06±0.05	0.33±0.03#	0.65±0.08#
	0.85	0.86±0.05	0.45±0.05#	0.94±0.09#

* p<0.05 and

# p<0.001 vs. baseline.

§ p<0.05 vs. 0.6;

† p<0.05 and

‡ p<0.005 vs. 0.85.

### Blood pressure, heart rate, carotid blood flow and cortical blood flow

**Figure 1:**
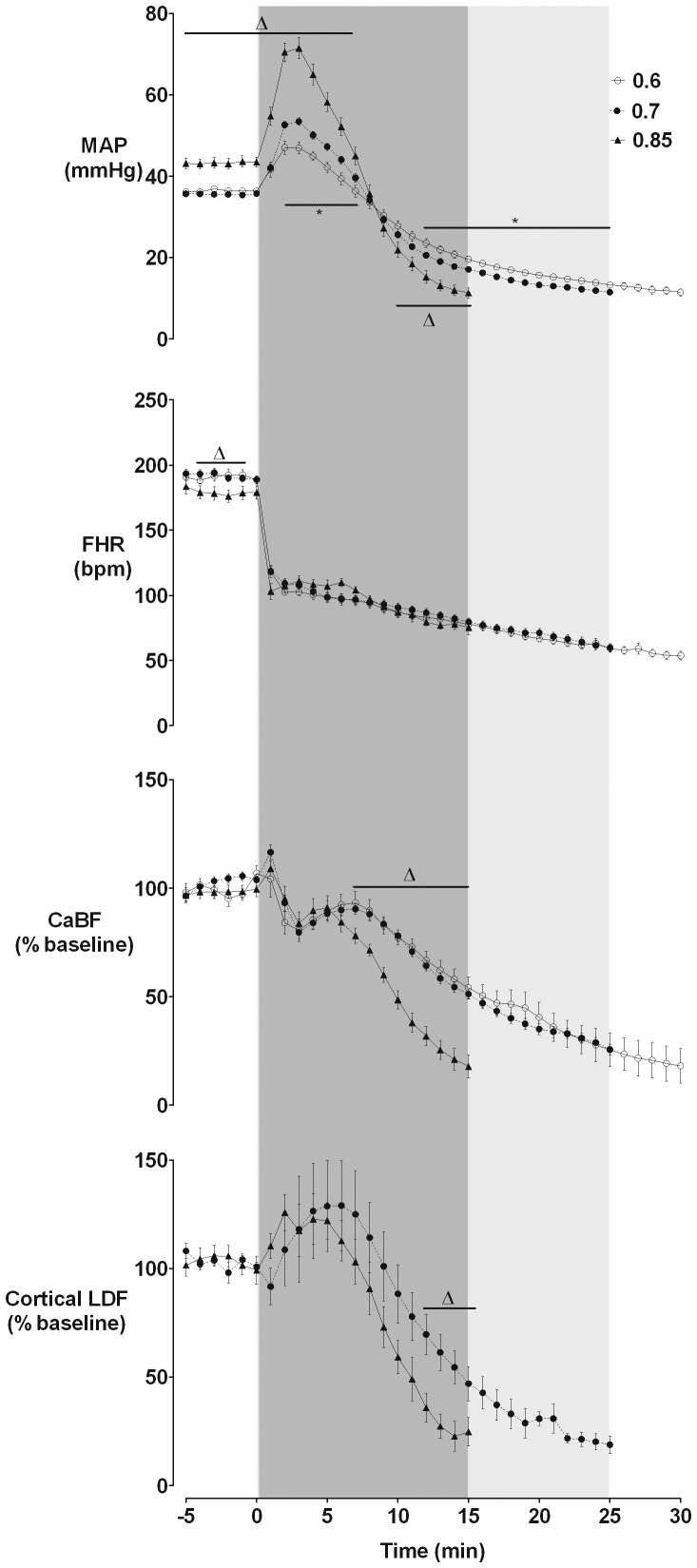
Changes in mean arterial pressure (MAP), fetal heart rate (FHR), carotid artery blood flow (CaBF) and cortical laser Doppler flow (LDF) during occlusion. Data are minute mean±S.E.M. Δ: p<0.05 for 0.85 vs. 0.6 and 0.7 groups; *: p<0.05 for 0.6 vs. 0.7 groups.

Occlusion was associated with an initial increase in MAP, followed by a rapid fall below baseline values and ultimately with profound hypotension at all ages. MAP was significantly higher in the 0.85 group at baseline (43.3±1.3 vs. 35.6±0.6 and 36.4±0.6 mmHg in the 0.6 and 0.7 ga groups respectively, p<0.05) and for the first 6 minutes of occlusion compared to 0.6 and 0.7 ga fetuses (p<0.05). MAP was also significantly higher over the first 2–7 min in the 0.7 ga compared to the 0.6 ga group (p<0.05). The onset of hypotension occurred earlier with increasing gestation (Figure 1). MAP fell significantly below baseline at 8 min the 0.85 ga group, and 9 min in the 0.6 and 0.7 ga groups. FHR was significantly lower at baseline in the 0.85 ga group compared to the 0.6 and 0.7 ga groups (178±5 bpm vs. 191±3 bpm in both 0.6 and 0.7 ga groups, p<0.05). Occlusion was associated with rapid bradycardia followed by a similar gradual further fall in all groups.

CaBF did not change significantly overall in the first 6 min after the onset of occlusion in any group, followed by a progressive fall after the onset of arterial hypotension. There were no differences in CaBF between the 0.6 and 0.7 ga groups. CaBF in 0.85 ga fetuses was significantly lower compared to 0.6 and 0.7 ga from 7–15 min (p<0.05). Cortical laser Doppler flow in 0.85 and 0.7 fetuses was highly correlated with CaBF (within subjects regression R^2^ = 0.62, p<0.0001), and overall showed a highly similar pattern. However, in contrast with CaBF, over the first 8 min there was a modest increase in laser Doppler flow (p<0.05, maximal at 4 min, with no independent effect of gestational age), which resolved to baseline values after 6 min. This was followed by a progressive fall after the onset of hypotension, which was more rapid at 0.85 than 0.7 ga.

### EEG and impedance

**Figure 2:**
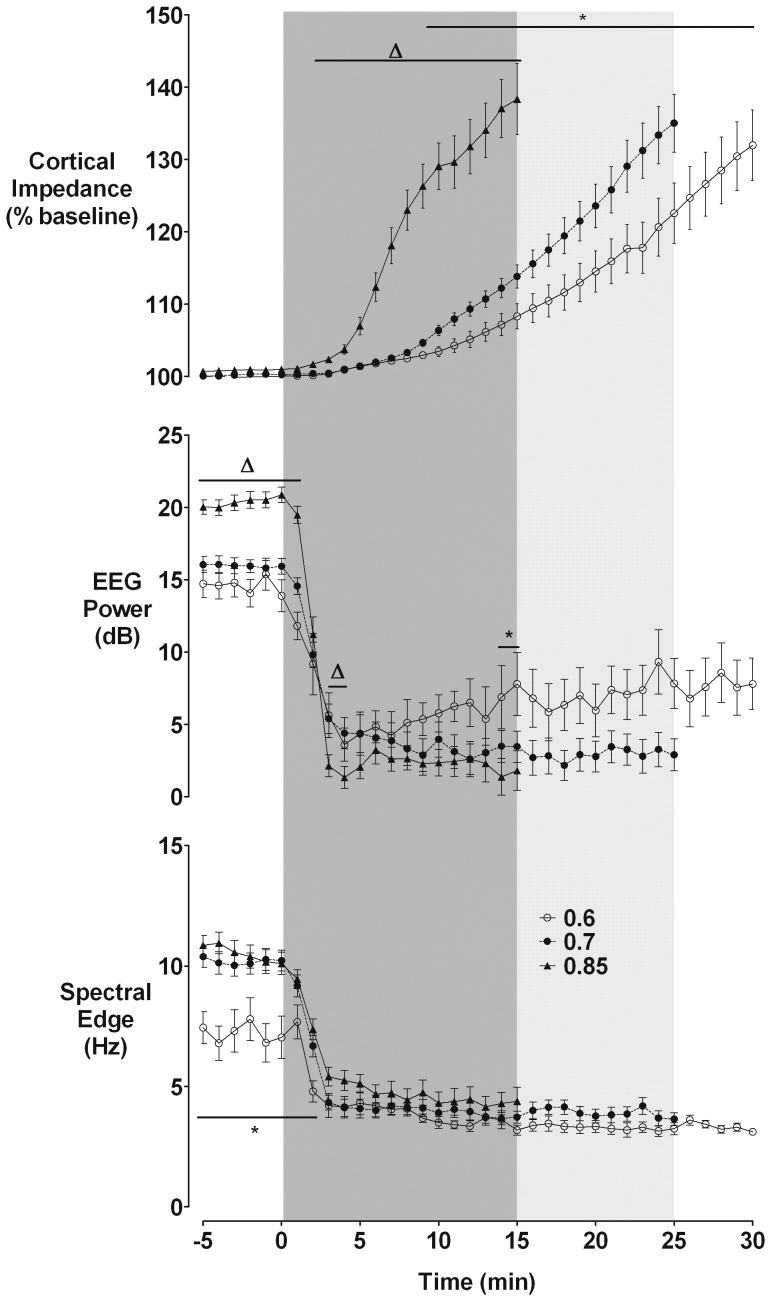
Changes in EEG power, spectral edge frequency, and cortical impedance during occlusion. Data are minute mean±S.E.M. Δ: p<0.05 for 0.85 vs. 0.6 and 0.7 groups; *: p<0.05 for 0.6 vs. 0.7 groups.

EEG power was significantly higher at baseline in the 0.85 group compared to the 0.7 and 0.6 ga groups (20.4±0.5 vs. 15.9±0.5 and 14.5±0.9 dB respectively, p<0.05, Figure 2). Occlusion was associated with rapid suppression of EEG activity in all groups, with the greatest fall in the 0.85 ga group (EEG power at 2 min of occlusion: 2.1±0.8 dB vs 5.4±1.0 dB in the 0.7 ga and 5.6±1.5 dB in the 0.6 ga groups, p<0.05). There were no significant differences between the 0.6 and 0.7 ga groups. Spectral edge was significantly lower at baseline in the 0.6 ga compared to the 0.7 ga and 0.85 ga groups (7.1±0.7 Hz vs. 10.1±0.4 Hz and 10.4±0.4 Hz respectively, p<0.05). All groups showed a rapid suppression of spectral edge frequency with no difference between groups during occlusion. Cortical impedance showed a progressive increase in all groups from several min after the start of occlusion. The relative rise in cortical impedance increased with increasing gestational age; the 0.7 group was significantly greater than the 0.6 group from 9 min (p<0.05) and the 0.85 group was significantly higher than the 0.6 and 0.7 group from 2–15 min (p<0.05). At 10 min of occlusion there was a clear maturation dependent difference in impedance (103±1%, 106±1%, and 129±3%, for 0.6, 0.7, and 0.85 ga respectively, p<0.05). The final maxima, at the end of occlusion, were similar between groups (132±4%, 135±4%, and 138±5%).

### Near-infrared spectroscopy

**Figure 3:**
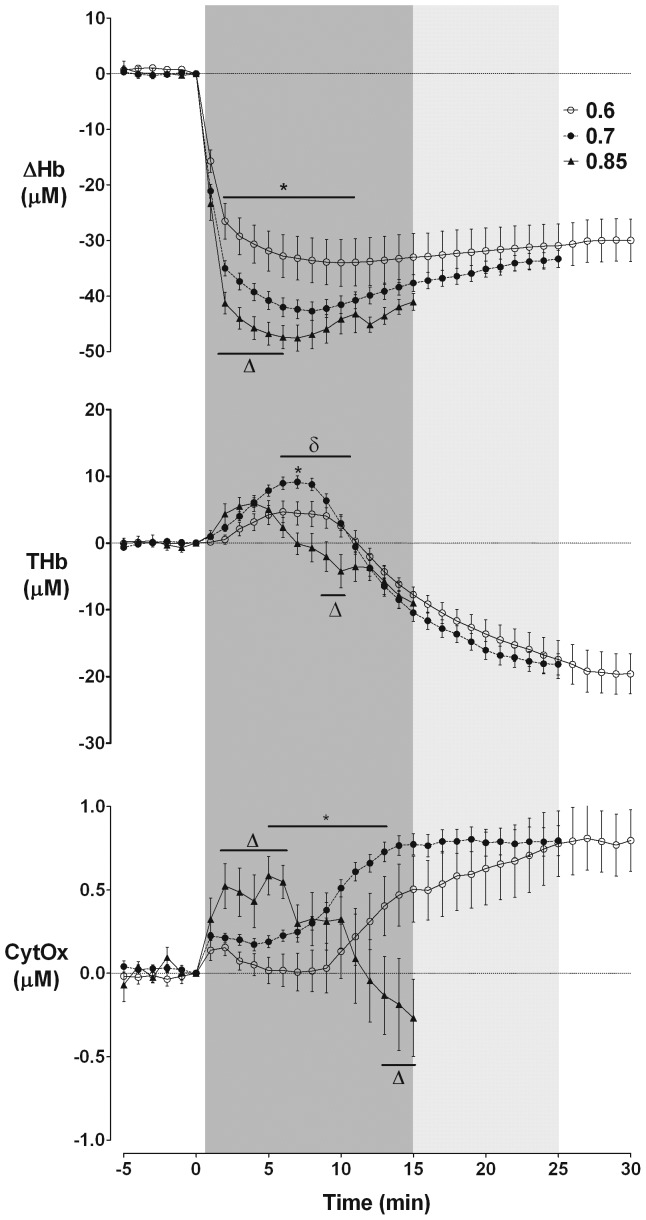
Concentration changes in ΔHb (HbO_2_-Hb), THb (HbO_2_+Hb) and oxidized cytochrome oxidase (CytOx) during occlusion. Data are one minute mean±S.E.M. Δ: p<0.05 for 0.85 vs. 0.6 and 0.7 groups; o: p<0.05 for 0.6 vs. 0.7 and 0.85 groups; *: p<0.05 for 0.6 vs. 0.7 groups; δ: p<0.05 for 0.7 vs. 0.85 groups.

Occlusion was associated with a rapid, profound fall in ΔHb. This fall was greater with increasing gestation (Figure 3), reaching a nadir of −34.0±4.2 µM in the 0.6 group, −42.7±1.7 µM in the 0.7 group, and −47.6±2.4 µM in the 0.85 group (p<0.05). After the nadir there was an apparent increase in ΔHb over the remainder of occlusion in all groups, mediated by a proportionately greater fall in Hb than HbO_2_; HbO_2_ did not increase (data not shown). THb initially increased during the compensation phase with a greater rise with increasing gestation, reaching a maxima of 4.7±1.6 µM at 6 min in the 0.6 group, 9.2±0.9 µM at 7 min in the 0.7 group, and 6.0±1.2 µM at 4 min in the 0.85 group (p<0.05, Figure 3). This was followed by a fall in THb in all groups; this fall began earlier in the 0.85 group and was significantly lower than the preterm groups from 6–10 min (p<0.05). Subsequent THb fell in all three groups in parallel, with similar values from around 11 min until the end of their respective occlusion (Figure 3).

Occlusion was associated with an initial increase in oxidized CytOx in all groups (p<0.05, Figure 3). The increase was greater in the 0.85 group than the 0.6 and 0.7 groups from 2–6 min (p<0.05). There was then a progressive rise over the remainder of occlusion in both 0.6 and 0.7 groups, which was initially greater in the 0.7 group (p<0.05, 5–13 min). In contrast, the 0.85 group showed a marked fall after approximately 7 min for the remainder of occlusion, and was significantly lower than both 0.6 and 0.7 groups from 13 min (p<0.05).

## Discussion

The present study shows that during profound asphyxia induced by complete umbilical cord occlusion in fetal sheep, greater maturity was associated with a more rapid rise in oxidized CytOx and greater suppression of cortical EEG activity, followed by markedly more rapid rise in cerebral impedance, indicating more rapid onset of neural depolarization and cell swelling. These findings strongly support the hypothesis that term fetuses are much more dependent on aerobic metabolism during periods of severe hypoxia than earlier in gestation. Unexpectedly, after the initial dramatic increase in oxidized CytOx near-term fetuses then showed a progressive loss of oxidized CytOx to below baseline levels, in contrast with a slow continued rise in the preterm fetuses. The mechanisms of the late fall are unknown, but as reviewed below, this pattern is broadly consistent with reports of a marked fall in oxidized CytOx during hypoxia-ischemia in postnatal animals and during dense cerebral ischemia in the fetal sheep [25], [26], [43]–[45]. Thus, speculatively it may reflect either greater cortical injury in near-term fetuses, or maturational changes in the response of the mitochondria.

Umbilical cord occlusion was associated with a rapid, profound fall in intracerebral oxygenation, as shown by a sustained fall in ΔHb on NIRS [27], with initial, rapid bradycardia and hypertension at all ages for approximately the first 7 min. This was followed by progressive hypotension, similarly to previous studies [2], [46]–[48]. The greater initial net fall in ΔHb in near-term fetuses supports the hypothesis of greater oxygen consumption with advancing gestation in fetal life. During this initial ‘compensation’ phase, before hypotension developed, oxidized CytOx rose rapidly, with the largest initial rise in near-term fetuses. The early increase was maintained throughout this compensation phase in the 0.85 and 0.7 gestation fetuses but, remarkably, returned to approximately baseline levels in the most immature, 0.6 gestation group. Consistent with the hypothesis that this increase indicated greater depletion of high energy metabolites (such as ATP and PCr), fetal cortical EEG power and frequency fell more in near-term fetuses. The fall in EEG power in particular was greater and faster in near-term fetuses, and reached a lower absolute nadir at 3 and 4 min of occlusion.

Cortical impedance rose earlier and much more rapidly near-term than in either the 0.7 or 0.65 gestation preterm fetuses. The impedance of a tissue rises concomitantly as cells depolarize and fluid shifts from the extracellular to the intracellular space causing cell swelling [28], and thus these data indicate earlier onset of neural depolarization and cell swelling with greater maturity. It is striking that impedance increased substantially more in the near-term fetuses before the onset of hypotension, indicating earlier onset of cortical depolarization. The initial rapid EEG suppression before the onset of cytotoxic edema is actively mediated by adenosine [17], and accumulation of other neuroinhibitors such as gamma amino butyric acid [49], [50], noradrenaline [51] and allopregnanalone [52]. Thus, for example, adenosine A1 receptor blockade during acute asphyxia in near-term fetal sheep, or ischemia in adult rats was associated with delayed onset of EEG suppression [17], [18], followed by more rapid onset of cortical depolarization and greater neural injury. Thus, the present data suggest that near-term fetal sheep are able to suppress EEG activity more at the onset of anoxia/hypoxia than preterm fetuses, and yet are less able to delay the onset of neural depolarization. Since depolarization is an essential contributor to hypoxic-ischemic brain injury this strongly supports the concept that greater maturity is associated with loss of neural tolerance to severe hypoxia, independent of cardiac compromise.

Despite the marked initial hypertension, which was greatest near-term, carotid blood flow remained relatively constant at all ages. This finding is in contrast with increased blood flow during moderate hypoxia or asphyxia [53], [54]. In part, this likely reflects a net redistribution of blood flow within the brain during asphyxia to subcortical structures, as measured by microspheres in the fetal sheep [16], [55], piglet [56] and newborn lamb [57]. Alternatively, carotid blood flow includes a significant proportion of extracerebral tissues [32] and thus a larger fraction of carotid flow perfusing non-brain tissues such as the face and scalp might be diverted to the brain during severe hypoxia than was evident from carotid blood flow measurements. Previous microsphere data suggest that severe asphyxia in utero is associated with either a small overall increase [16], or no change in cerebral blood flow [55]. Supporting a small net increase in perfusion, in the present study there was a modest, transient increase in both cortical blood flow (laser Doppler) and THb, that was greatest in near-term fetuses. Although THb is not a direct measure of cerebral perfusion, the increase broadly paralleled the changes in cortical blood flow and is in agreement with the previous finding that cerebral blood flow increases much more during induced hypoxia in near-term than preterm fetuses [58].

Progressive hypotension developed from approximately 9 minutes of occlusion. The onset of hypotension corresponded closely with the onset of both global and local (cortical) hypoperfusion at all ages. This is highly consistent with the lower limit of cerebral autoregulation being just below baseline blood pressure [59], and with evidence of impaired autoregulation during partial asphyxia in the near-term fetal sheep [60] and inhalational hypoxia in the lamb [61]. In both preterm groups in the present study, the onset of hypotension was associated with a further, delayed rise in oxidized CytOx, followed by linear increases in cortical impedance. Similarly, in newborn (postnatal day 7, P7) rats, when brain development is relatively preterm [62], hypoxia-ischemia was associated with an initial reduction of CytOx, followed by delayed oxidation to above baseline levels once ATP levels fell to their nadir [43]. The rises were earlier and more rapid at 0.7 than 0.6 gestation in the present study, despite similar relative falls in blood pressure and carotid blood flow, strongly denoting reduced neural tolerance to anoxia with greater maturity. Supporting this interpretation, the two groups reached essentially identical maxima for oxidized CytOx, suggesting that this late increase in oxidized CytOx reflects progressive loss of production of reducing equivalents due to loss of residual anerobic metabolism. Potentially, active inhibition of mitochondrial function might also contribute to part of the rise, since nitric oxide, for example, is known to inhibit respiratory complexes I and IV [63], and there is evidence that the nitric oxide synthases are more abundant in the immature brain in both sheep and in post-mortem human tissue [64], [65].

In contrast, after the onset of hypotension the 0.85 gestation fetuses showed a profound and unexpected fall in oxidized CytOx. This is broadly consistent with the majority of postnatal studies of hypoxia-ischemia, including adults rats [25], [26], deep-hypothermic circulatory arrest in the newborn [25] and adult pig [44], and human infants [45], hypotension induced by blood withdrawal during hypoxia in lambs and severe cerebral ischemia in the near-term fetus [66], [67]. In contrast, in near-term and newborn lambs moderate hypoxia was associated with an increase in oxidized CytOx [67], [68]. It is intriguing to note that in previous studies showing an increase in oxidized CytOx cortical injury was not seen [17], [27], [67], [68]. Conversely, in fetal sheep cerebral ischemia leading to severe cortical injury was associated with a terminal fall in oxidized CytOx, although the precise time course is unknown [69], [70].

Despite the apparent fall in measured oxidized CytOx in near-term fetuses, it seems rather improbable that there can been a true shift to more reduced CytOx during profound anoxia, with highly limited substrate delivery due to hypotension and hypoglycemia. A more plausible hypothesis is that it reflects a combination of two factors, the increase in brain size with age, and increasing cortical susceptibility to injury. First, in the late gestation fetal sheep brain weight doubles approximately every fortnight. The exact area interrogated by near-infrared light is not clear, but it is likely that as the overall size of the brain increases, the cortex would contribute an increasing fraction. Second, a fall in the CytOx signal must reflect a loss of oxidized cytochrome c oxidase; if it is not related to enzymatic reduction, then there could be exposure to the reducing environment of the cytosol either through opening of the mitochondrial permeability transition pore [71], or frank structural damage of the mitochondria. There is evidence for a maturation dependent change in the influence of the mitochondrial permeability transition pore on injury, with apparently little role in immature mice compared to older mice [72]. Further, in vitro, prolonged oxygen-glucose deprivation in rat cortical slices also led to a fall in oxidized CytOx [73], and in blood-free perfused rat brain, cellular ATP only started to fall when CytOx became less oxidized [74]. Thus these data suggest that a fall in oxidized CytOx represents a transition to mitochondrial injury, and may be a useful intra-insult biomarker of injury.

Potentially, the fall in Hb in the brain during occlusion could have influenced the changes in CytOx because of the much greater spectral absorption of Hb compared to CytOx [75]. However, this speculation is not consistent with our observation that oxidized CytOx changes were unrelated to the very large changes in hemoglobin signals. In the first 5 min after occlusion THb increased above baseline similarly in all groups despite markedly larger increase in oxidized CytOx in the near-term fetuses. Although subsequently hypotension developed more rapidly in the near-term fetuses, THb fell at a similar rate at all ages after the onset of hypotension, and the magnitude of the final fall in THb was greatest in the preterm fetuses. Since this parameter is an accurate index of total cerebral blood volume determined by radiolabeling in the piglet [40], this strongly infers that oxidized CytOx changed independently of cerebral blood volume.

In conclusion, the present study demonstrates a maturation dependent change in the mitochondrial response to profound asphyxia in fetal sheep, consistent with an intrinsic loss of neural tolerance to severe hypoxia-ischemia. Near-term fetuses showed markedly more rapid increase in oxidation of CytOx, followed by more rapid onset of cytotoxic edema, well before the onset of systemic hypotension or hypoperfusion. During the subsequent progressive cardiovascular decompensation, near-term fetuses showed a more rapid fall blood pressure and carotid and cortical blood flow than preterm fetuses, with accelerated cytotoxic edema, but a paradoxical loss of oxidized cytochrome oxidase. This pattern strongly supports the hypothesis of increasing dependence on aerobic metabolism towards term, independent of the loss of cardiac tolerance to anoxia.

## References

[1] Dawes GS, Mott JC, Shelley HJ (1959). The importance of cardiac glycogen for the maintenance of life in foetal lambs and newborn animals during anoxia.. J Physiol.

[2] Wassink G, Bennet L, Booth LC, Jensen EC, Wibbens B (2007). The ontogeny of hemodynamic responses to prolonged umbilical cord occlusion in fetal sheep.. J Appl Physiol.

[3] Mallard EC, Williams CE, Johnston BM, Gluckman PD (1994). Increased vulnerability to neuronal damage after umbilical cord occlusion in fetal sheep with advancing gestation.. Am J Obstet Gynecol.

[4] Mallard EC, Gunn AJ, Williams CE, Johnston BM, Gluckman PD (1992). Transient umbilical cord occlusion causes hippocampal damage in the fetal sheep.. Am J Obstet Gynecol.

[5] Ley D, Oskarsson G, Bellander M, Hernandez-Andrade E, Lingman G (2004). Different responses of myocardial and cerebral blood flow to cord occlusion in exteriorized fetal sheep.. Pediatr Res.

[6] Wibbens B, Westgate JA, Bennet L, Roelfsema V, de Haan HH (2005). Profound hypotension and associated ECG changes during prolonged cord occlusion in the near term fetal sheep.. Am J Obstet Gynecol.

[7] Keunen H, Blanco CE, van Reempts JL, Hasaart TH (1997). Absence of neuronal damage after umbilical cord occlusion of 10, 15, and 20 minutes in midgestation fetal sheep.. Am J Obstet Gynecol.

[8] George S, Gunn AJ, Westgate JA, Brabyn C, Guan J (2004). Fetal heart rate variability and brainstem injury after asphyxia in preterm fetal sheep.. Am J Physiol Regul Integr Comp Physiol.

[9] Gleason CA, Hamm C, Jones MD (1989). Cerebral blood flow, oxygenation, and carbohydrate metabolism in immature fetal sheep in utero.. Am J Physiol.

[10] Jensen A, Berger R (1991). Fetal circulatory responses to oxygen lack.. J Dev Physiol.

[11] Berger R, Jensen A, Krieglstein J, Steigelmann JP (1991). Cerebral energy metabolism in guinea pig fetuses during development.. J Dev Physiol.

[12] Duffy TE, Kohle SJ, Vannucci RC (1975). Carbohydrate and energy metabolism in perinatal rat brain: relation to survival in anoxia.. J Neurochem.

[13] Sylvia AL, Seidler FJ, Slotkin TA (1989). Effect of transient hypoxia on oxygenation of the developing rat brain: relationships among haemoglobin saturation, autoregulation of blood flow and mitochondrial redox state.. J Dev Physiol.

[14] Azzopardi D, Wyatt JS, Hamilton PA, Cady EB, Delpy DT (1989). Phosphorus metabolites and intracellular pH in the brains of normal and small for gestational age infants investigated by magnetic resonance spectroscopy.. Pediatr Res.

[15] Astrup J (1982). Energy-requiring cell functions in the ischemic brain. Their critical supply and possible inhibition in protective therapy.. J Neurosurg.

[16] Kaneko M, White S, Homan J, Richardson B (2003). Cerebral blood flow and metabolism in relation to electrocortical activity with severe umbilical cord occlusion in the near-term ovine fetus.. Am J Obstet Gynecol.

[17] Hunter CJ, Bennet L, Power GG, Roelfsema V, Blood AB (2003). Key neuroprotective role for endogenous adenosine A1 receptor activation during asphyxia in the fetal sheep.. Stroke.

[18] Ilie A, Ciocan D, Zagrean AM, Nita DA, Zagrean L (2006). Endogenous activation of adenosine A(1) receptors accelerates ischemic suppression of spontaneous electrocortical activity.. Journal of Neurophysiology.

[19] Mortola JP (2004). Implications of hypoxic hypometabolism during mammalian ontogenesis.. Respir Physiol Neurobiol.

[20] Gunn AJ, Quaedackers JS, Guan J, Heineman E, Bennet L (2001). The premature fetus: not as defenseless as we thought, but still paradoxically vulnerable?. Dev Neurosci.

[21] Dieni S, Rees S (2003). Dendritic morphology is altered in hippocampal neurons following prenatal compromise.. J Neurobiol.

[22] Jobsis FF (1977). Noninvasive, infrared monitoring of cerebral and myocardial oxygen sufficiency and circulatory parameters.. Science.

[23] Moroz T, Banaji M, Robertson NJ, Cooper CE, Tachtsidis I (2012). Computational modelling of the piglet brain to simulate near-infrared spectroscopy and magnetic resonance spectroscopy data collected during oxygen deprivation.. Journal of the Royal Society Interface Epub Jan 25.

[24] Boushel R, Piantadosi CA (2000). Near-infrared spectroscopy for monitoring muscle oxygenation.. Acta Physiol Scand.

[25] Springett RJ, Wylezinska M, Cady EB, Hollis V, Cope M (2003). The oxygen dependency of cerebral oxidative metabolism in the newborn piglet studied with 31P NMRS and NIRS.. Adv Exp Med Biol.

[26] Matsumoto H, Oda T, Hossain MA, Yoshimura N (1996). Does the redox state of cytochrome aa3 reflect brain energy level during hypoxia? Simultaneous measurements by near infrared spectrophotometry and 31P nuclear magnetic resonance spectroscopy.. Anesth Analg.

[27] Bennet L, Roelfsema V, Dean J, Wassink G, Power GG (2007). Regulation of cytochrome oxidase redox state during umbilical cord occlusion in preterm fetal sheep.. Am J Physiol Regul Integr Comp Physiol.

[28] Williams CE, Gunn A, Gluckman PD (1991). Time course of intracellular edema and epileptiform activity following prenatal cerebral ischemia in sheep.. Stroke.

[29] van Bel F, Roman C, Klautz RJ, Teitel DF, Rudolph AM (1994). Relationship between brain blood flow and carotid arterial flow in the sheep fetus.. Pediatr Res.

[30] Bennet L, Rossenrode S, Gunning MI, Gluckman PD, Gunn AJ (1999). The cardiovascular and cerebrovascular responses of the immature fetal sheep to acute umbilical cord occlusion.. J Physiol.

[31] Gonzalez H, Hunter CJ, Bennet L, Power GG, Gunn AJ (2005). Cerebral oxygenation during post-asphyxial seizures in near-term fetal sheep.. J Cereb Blood Flow Metab.

[32] Dunnihoo DR, Quilligan EJ (1973). Carotid blood flow distribution in the in utero sheep fetus.. American Journal of Obstetrics and Gynecology.

[33] McIntosh GH, Baghurst KI, Potter BJ, Hetzel BS (1979). Foetal brain development in the sheep.. Neuropathol Appl Neurobiol.

[34] Barlow RM (1969). The foetal sheep: morphogenesis of the nervous system and histochemical aspects of myelination.. J Comp Neurol.

[35] Bennet L, Roelfsema V, Pathipati P, Quaedackers J, Gunn AJ (2006). Relationship between evolving epileptiform activity and delayed loss of mitochondrial activity after asphyxia measured by near-infrared spectroscopy in preterm fetal sheep.. J Physiol.

[36] Lawler FH, Brace RA (1982). Fetal and maternal arterial pressures and heart rates: histograms, correlations, and rhythms.. Am J Physiol.

[37] Williams CE, Gunn AJ, Mallard C, Gluckman PD (1992). Outcome after ischemia in the developing sheep brain: an electroencephalographic and histological study.. Ann Neurol.

[38] Reynolds EO, Wyatt JS, Azzopardi D, Delpy DT, Cady EB (1988). New non-invasive methods for assessing brain oxygenation and haemodynamics.. Br Med Bull.

[39] Wyatt JS, Cope M, Delpy DT, Richardson CE, Edwards AD (1990). Quantitation of cerebral blood volume in human infants by near-infrared spectroscopy.. J Appl Physiol.

[40] Barfield CP, Yu VY, Noma O, Kukita J, Cussen LJ (1999). Cerebral blood volume measured using near-infrared spectroscopy and radiolabels in the immature lamb brain.. Pediatr Res.

[41] Brun NC, Moen A, Borch K, Saugstad OD, Greisen G (1997). Near-infrared monitoring of cerebral tissue oxygen saturation and blood volume in newborn piglets.. Am J Physiol.

[42] Bland JM, Altman DG (1995). Calculating correlation coefficients with repeated observations: Part 1–Correlation within subjects.. Bmj.

[43] Yager JY, Brucklacher RM, Vannucci RC (1996). Paradoxical mitochondrial oxidation in perinatal hypoxic-ischemic brain damage.. Brain Res.

[44] Gagnon RE, Macnab AJ, Gagnon FA, Leblanc JG (2005). Brain, spine, and muscle cytochrome Cu-A redox patterns of change during hypothermic circulatory arrest in swine.. Comp Biochem Physiol A Mol Integr Physiol.

[45] du Plessis AJ, Newburger J, Jonas RA, Hickey P, Naruse H (1995). Cerebral oxygen supply and utilization during infant cardiac surgery.. Ann Neurol.

[46] Barcroft J (1946). Researches in prenatal life.. London and Oxford: Blackwell Scientific Publications Ltd.

[47] Itskovitz J, Rudolph AM (1982). Denervation of arterial chemoreceptors and baroreceptors in fetal lambs in utero.. Am J Physiol.

[48] Bartelds B, van Bel F, Teitel DF, Rudolph AM (1993). Carotid, not aortic, chemoreceptors mediate the fetal cardiovascular response to acute hypoxemia in lambs.. Pediatr Res.

[49] Tan WK, Williams CE, During MJ, Mallard CE, Gunning MI (1996). Accumulation of cytotoxins during the development of seizures and edema after hypoxic-ischemic injury in late gestation fetal sheep.. Pediatr Res.

[50] Nilsson GE, Lutz PL (2004). Anoxia tolerant brains.. J Cereb Blood Flow Metab.

[51] Dean JM, George S, Naylor AS, Mallard C, Gunn AJ (2008). Partial neuroprotection with low-dose infusion of the 2-adrenergic receptor agonist clonidine after severe hypoxia in preterm fetal sheep.. Neuropharmacology.

[52] Yawno T, Yan EB, Walker DW, Hirst JJ (2007). Inhibition of neurosteroid synthesis increases asphyxia-induced brain injury in the late gestation fetal sheep.. Neuroscience.

[53] Giussani DA, Spencer JA, Moore PJ, Bennet L, Hanson MA (1993). Afferent and efferent components of the cardiovascular reflex responses to acute hypoxia in term fetal sheep.. J Physiol.

[54] Ball RH, Parer JT, Caldwell LE, Johnson J (1994). Regional blood flow and metabolism in ovine fetuses during severe cord occlusion.. Am J Obstet Gynecol.

[55] Jensen A, Hohmann M, Kunzel W (1987). Dynamic changes in organ blood flow and oxygen consumption during acute asphyxia in fetal sheep.. J Dev Physiol.

[56] Goplerud JM, Wagerle LC, Delivoria-Papadopoulos M (1989). Regional cerebral blood flow response during and after acute asphyxia in newborn piglets.. J Appl Physiol.

[57] Lou HC, Tweed WA, Davies JM (1985). Preferential blood flow increase to the brain stem in moderate neonatal hypoxia: reversal by naloxone.. Eur J Pediatr.

[58] Gleason CA, Hamm C, Jones MD (1990). Effect of acute hypoxemia on brain blood flow and oxygen metabolism in immature fetal sheep.. Am J Physiol.

[59] Papile LA, Rudolph AM, Heymann MA (1985). Autoregulation of cerebral blood flow in the preterm fetal lamb.. Pediatr Res.

[60] Johnson GN, Palahniuk RJ, Tweed WA, Jones MV, Wade JG (1979). Regional cerebral blood flow changes during severe fetal asphyxia produced by slow partial umbilical cord compression.. Am J Obstet Gynecol.

[61] Tweed A, Cote J, Lou H, Gregory G, Wade J (1986). Impairment of cerebral blood flow autoregulation in the newborn lamb by hypoxia.. Pediatr Res.

[62] Romijn HJ, Hofman MA, Gramsbergen A (1991). At what age is the developing cerebral cortex of the rat comparable to that of the full-term newborn human baby?. Early Hum Dev.

[63] Brown GC (1997). Nitric oxide inhibition of cytochrome oxidase and mitochondrial respiration: implications for inflammatory, neurodegenerative and ischaemic pathologies.. Mol Cell Biochem.

[64] Wood CE, Chen GF, Keller-Wood M (2005). Expression of nitric oxide synthase isoforms is reduced in late-gestation ovine fetal brainstem.. Am J Physiol Regul Integr Comp Physiol.

[65] Downen M, Zhao ML, Lee P, Weidenheim KM, Dickson DW (1999). Neuronal nitric oxide synthase expression in developing and adult human CNS.. J Neuropathol Exp Neurol.

[66] Marks KA, Mallard EC, Roberts I, Williams CE, Sirimanne ES (1996). Delayed vasodilation and altered oxygenation after cerebral ischemia in fetal sheep.. Pediatr Res.

[67] Shadid M, Hiltermann L, Monteiro L, Fontijn J, van Bel F (1999). Near infrared spectroscopy-measured changes in cerebral blood volume and cytochrome aa3 in newborn lambs exposed to hypoxia and hypercapnia, and ischemia: a comparison with changes in brain perfusion and O2 metabolism.. Early Hum Dev.

[68] Newman JP, Peebles DM, Harding SR, Springett R, Hanson MA (2000). Hemodynamic and metabolic responses to moderate asphyxia in brain and skeletal muscle of late-gestation fetal sheep.. J Appl Physiol.

[69] Reddy K, Mallard C, Guan J, Marks K, Bennet L (1998). Maturational change in the cortical response to hypoperfusion injury in the fetal sheep.. Pediatr Res.

[70] Fraser M, Bennet L, Gunning M, Williams CE, Gluckman PD (2005). Cortical electroencephalogram suppression is associated with post-ischemic cortical injury in 0.65 gestation fetal sheep.. Brain Res Dev Brain Res.

[71] Andrabi SA, Sayeed I, Siemen D, Wolf G, Horn TF (2004). Direct inhibition of the mitochondrial permeability transition pore: a possible mechanism responsible for anti-apoptotic effects of melatonin.. Faseb J.

[72] Wang X, Carlsson Y, Basso E, Zhu C, Rousset CI (2009). Developmental shift of cyclophilin D contribution to hypoxic-ischemic brain injury.. J Neurosci.

[73] Nishidate I, Yoshida K, Sato M (2010). Changes in optical properties of rat cerebral cortical slices during oxygen glucose deprivation.. Applied optics.

[74] Matsunaga A, Nomura Y, Kuroda S, Tamura M, Nishihira J (1998). Energy-dependent redox state of heme a+a3 and copper of cytochrome oxidase in perfused rat brain in situ.. Am J Physiol.

[75] Wray S, Cope M, Delpy DT, Wyatt JS, Reynolds EO (1988). Characterization of the near infrared absorption spectra of cytochrome aa3 and haemoglobin for the non-invasive monitoring of cerebral oxygenation.. Biochim Biophys Acta.

